# Neuronal networks provide rapid neuroprotection against spreading toxicity

**DOI:** 10.1038/srep33746

**Published:** 2016-09-21

**Authors:** Andrew J. Samson, Graham Robertson, Michele Zagnoni, Christopher N. Connolly

**Affiliations:** 1 Division of Neuroscience, School of Medicine, University of Dundee, Dundee, DD1 9SY, Scotland, Uk; 2 Centre for Microsystems and Photonics, Department of Electronic and Electrical Engineering, University of Strathclyde, Glasgow, G1 1XW, Scotland, Uk.

## Abstract

Acute secondary neuronal cell death, as seen in neurodegenerative disease, cerebral ischemia (stroke) and traumatic brain injury (TBI), drives spreading neurotoxicity into surrounding, undamaged, brain areas. This spreading toxicity occurs *via* two mechanisms, synaptic toxicity through hyperactivity, and excitotoxicity following the accumulation of extracellular glutamate. To date, there are no fast-acting therapeutic tools capable of terminating secondary spreading toxicity within a time frame relevant to the emergency treatment of stroke or TBI patients. Here, using hippocampal neurons (DIV 15–20) cultured in microfluidic devices in order to deliver a localized excitotoxic insult, we replicate secondary spreading toxicity and demonstrate that this process is driven by GluN2B receptors. In addition to the modeling of spreading toxicity, this approach has uncovered a previously unknown, fast acting, GluN2A-dependent neuroprotective signaling mechanism. This mechanism utilizes the innate capacity of surrounding neuronal networks to provide protection against both forms of spreading neuronal toxicity, synaptic hyperactivity and direct glutamate excitotoxicity. Importantly, network neuroprotection against spreading toxicity can be effectively stimulated after an excitotoxic insult has been delivered, and may identify a new therapeutic window to limit brain damage.

During the development of the central nervous system, competition for synapse formation and early patterns of neuronal network activity are required for neurons to “fire together and wire together”, driving the formation of functional neuronal networks^1^,^2^,^3^. Once established, neuronal survival is conditional upon continued participation in network activity. However, following cerebral ischemia (stroke) or traumatic brain injury (TBI), synapsed neurons in the surrounding penumbral region are at high risk from spreading depolarizations^4^ and elevated extracellular glutamate released by cell lysis and transporter reversal^5^, leading to NMDA receptor dependent synaptic toxicity^5^,^6^,^7^ and excitotoxicity^5^,^7^,^8^,^9^,^10^. Paradoxically, the use of NMDA receptor antagonists as neuroprotectants actually exacerbates brain injury^11^,^12^ due to inhibition of essential pro-survival signaling that occurs through these receptors^10^,^13^,^14^. To date, successful protection of neurons against damage can be achieved by the use of preconditioning paradigms, where low-level stimulation^13^,^14^,^15^,^16^,^17^, or exercise^18^,^19^, can induce a neuroprotective state to subsequent larger insults. Unfortunately, the duration (days) of preconditioning required for neuroprotection to develop limits its clinical value.

Given that damage from a lesion does not spread uncontrollably to consume the entire brain, combined with our knowledge that synaptic neurotransmission can be protective^10^,^13^,^14^, it is reasonable to assume that neuronal networks may possess an innate capacity to restrict damage *in vivo*. However, a problem in studying spreading toxicity to naïve neurons *in vivo* is the difficulty in separating the initial lesion from its downstream consequences. We have achieved this separation using an *in vitro* model based on a microfluidic channel network, where multiple neuron populations, that are environmentally isolated but synaptically connected, are cultured and their microenvironment precisely manipulated^20^. Using this approach, we can isolate activity-dependent spreading toxicity from direct glutamate excitotoxicity and use this to model and investigate potential neuroprotective network activity.

## Results

### Functional synaptic communication between environmentally-isolated neuronal networks

**Figure 1:**
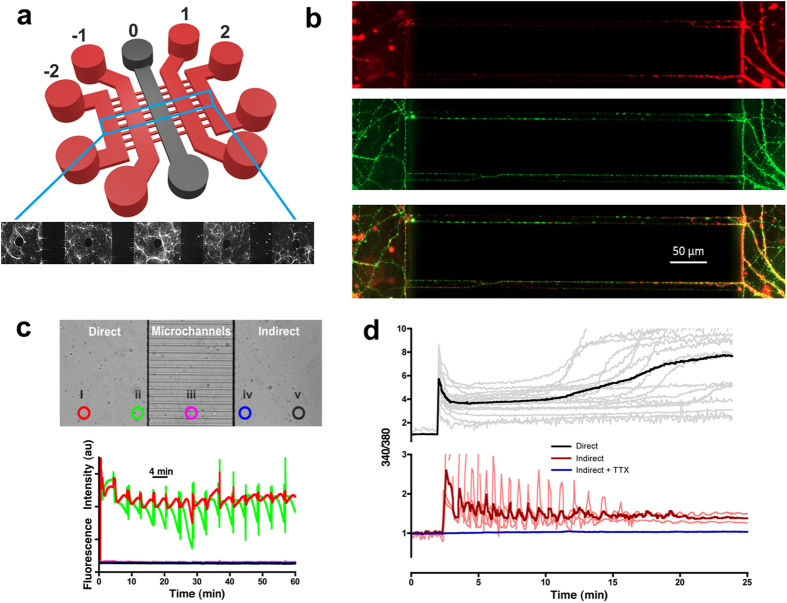
Functional synaptic communication between environmentally-isolated neuronal networks. **(a)** Schematic of the microfluidic device showing the five parallel culture chambers (−2, −1, 0, 1, 2). (**b**) Hippocampal neurons cultured in microfluidic devices labeled to distinguish dendrites (MAP2, red) and axons (tubulin, green). Scale bar = 50 μm. **(c)** Validation of microfluidic protocol for insult delivery. (*Top*) Light microscopy image of the microchannel barrier separating the insulted chamber (‘direct’) from the insult-free chamber (‘indirect’). Highlighted circles represent the points of fluorescein fluorescence monitoring. (*Bottom*) Fluorescent intensity profiles obtained from the five regions of interest (i–v) using the insult protocol detailed in Materials and Methods. No cross contamination of fluorescein was observed in any indirect chambers, whilst an almost constant fluorescence profile was achieved across the width of the insulted chamber (circle i). **(d)** A direct excitotoxic challenge (100 GG) leads to delayed Ca^2+^ deregulation (n = 4) and downstream (indirect), activity-dependent (+TTX, blue traces, n = 3) Ca^2+^ spiking activity (red traces, n = 3). Traces are representative of individual neuronal responses (light color) and the mean for all recorded cells (dark color). Experiments were performed using independent neuronal preparations.

In order to isolate secondary spreading toxicity from the primary excitotoxic insult, we adopted the use of a microfluidic system having five cell culture chambers serially interconnected by 500 μm long microchannels (Fig. 1a). Hippocampal neurons were cultured in each chamber and synaptically connected *via* axons traversing the microchannels (Fig. 1b). A protocol (detailed in Materials and Methods) was developed to ensure that, during exposure of an insult in the desired chamber, no cross-contamination occurred into surrounding chambers. To validate the protocol, a fluorescein suspension was added to the ‘direct’ insulted chamber (chamber ‘0’, Fig. 1a) and its diffusion monitored *via* epifluorescence microscopy across a field of view spanning the ‘direct’ and ‘indirect’ chambers at five different locations (Fig. 1c). The fluorescence signals show that an almost constant intensity is achieved at the site of delivery, with no fluorescence detected in the microchannels or surrounding chambers^20^.

Prior to microfluidic experimentation, we established the neuronal responses to excitotoxicity. Hippocampal neuron-seeded coverslips were exposed to various concentrations of glutamate (with glycine as a co-agonist) for 1 hour, and stained with Hoeschst-33342 (4.5 μM) and propidium iodide (20 μM) 24 hours later to determine the percentage of live/dead cells, respectively. Excitotoxicity to glutamate occurred at 100 μM (100 GG, 78.1 ± 17.83% cell death, P < 0.001), with 50 μM being subtoxic (50 GG, 35.45 ± 11.19% cell death, P > 0.05). As reported previously^15^, following an excitotoxic challenge (100 GG) to a single neuronal chamber in the microfluidic device, a rapid influx of Ca^2+^ is observed, followed by a prolonged raised basal level (the latent period) and ultimately to delayed Ca^2+^ deregulation (Fig. 1d, upper panel, black trace). Confirming neuronal communication exists between chambers, a consequential Ca^2+^ response with rapid spiking activity occurs in downstream chambers (Fig. 1d, lower panel, red trace). These downstream signals are abolished in the absence of action potential firing (tetrodotoxin, TTX, 0.5 μM) (Fig. 1d, lower panel, blue trace).

### Glutamate-induced spreading neurotoxicity through neuronal networks

**Figure 2:**
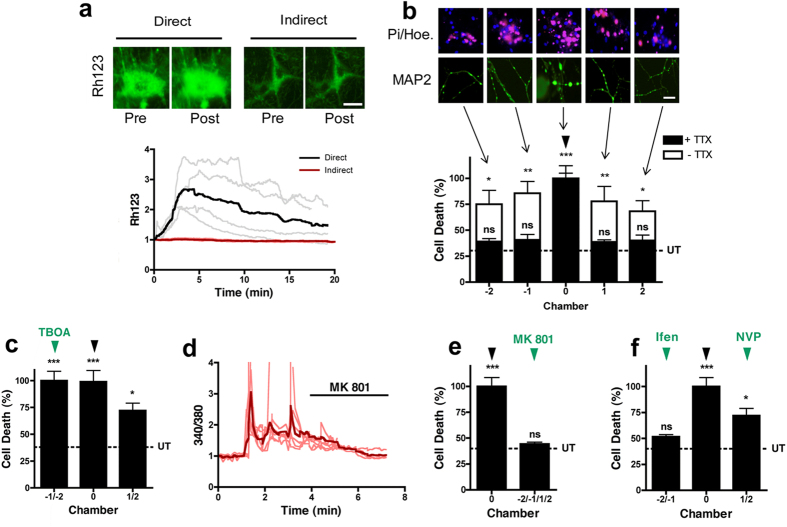
Glutamate-induced spreading neurotoxicity through neuronal networks. **(a)** Mitochondrial depolarization occurs in neurons directly challenged with excitotoxic glutamate), but this does not spread to downstream neurons. (*Top*) Representative images of rhodamine 123 fluorescence prior to and following a 100 GG application in chamber 0 (direct). (*Bottom*) Relative change in rhodamine 123 fluorescence in direct (100 GG, black trace) and indirect (red trace, n = 4) chambers. Traces are representative of individual neuronal responses (light color) and the mean for all recorded cells (dark color). **(b)** Delivery of excitotoxic glutamate to neurons in chamber 0, reveals an activity-dependent spreading toxicity. (*Top*) Representative PI/Hoechst (Hoe.) staining in each neuronal chamber following delivery of excitotoxic glutamate. (*Middle*) Representative MAP2 staining revealing spreading dendrotoxicity following localized excitotoxic glutamate. (*Bottom*) Quantitative change in cell death following delivery of excitotoxic glutamate (100 GG, 1 hour, black arrowhead) to neurons in chamber 0, in the absence (white bars, n = 6) and presence (black bars, n = 4) of TTX (0.5 μM). **(c)** Delivery of excitotoxic glutamate to neurons in chamber 0 in the presence of TFB-TBOA (0.1 μM) in chambers −2/−1 (n = 4). **(d)** Downstream Ca^2+^ spikes evoked by an indirect excitotoxic challenge are blocked by MK 801 (10 μM, n = 4). Traces are representative individual neuronal responses (light color) and the mean for all recorded cells (dark color). **(e)** Spreading toxicity to downstream chambers is abolished in the presence of downstream MK 801 (n = 4) or **(f)** Ifenprodil (10 μM, n = 3), but not NVP-AAM077 (0.5 μM, n = 3). Basal cell death is indicated by the dotted line (UT). Data are expressed as mean ± S.E.M (one-way ANOVA with post hoc Tukey’s test. ‘ns’ denotes p > 0.05, *denotes p < 0.05, **denotes p < 0.01, ***denotes p < 0.001, relative to control). Experiments were performed using independent neuronal preparations.

Mitochondrial depolarization, an early indicator of excitotoxicity^15^, occurs in the chamber directly exposed to 100 GG (Fig. 2a, direct), but despite the Ca^2+^ spikes present in the downstream neuronal chambers, mitochondrial depolarization does not spread (Fig. 2a, indirect). This suggests a localized excitotoxic insult evokes a spreading hyperactivity to a wider neuronal network^4^,^5^,^6^,^7^. To explore whether toxicity spreads in the absence of spreading extracellular glutamate, an excitotoxic insult (100 GG, 1 hour) was delivered to the central chamber only (Fig. 2b, chamber 0) and morphological changes (MAP2 staining) and cell death (propidium iodide) determined in all 5 chambers after 1 hour or 24 hours, respectively. As expected, extensive dendritic beading and neuronal cell death was observed in the directly exposed central chamber (62.84 ± 13.28% cell death, subsequently normalized to 100% for clarity). Dendrotoxicity and cell death spread from the central chamber to connected neurons in both adjacent (−1/+1) chambers (85.6 ± 19.78%/77.73 ± 25.1% respectively) and into both distal (−2/+2) chambers (75.15 ± 22.84%/68.35 ± 17.28%, respectively) (Fig. 2b, white bars), as compared to control saline-treated devices (UT, 30.44 ± 3.0%). In the absence of synaptic neurotransmission (TTX, 0.5 μM), spreading toxicity is abolished in all downstream chambers (−1/+1; 42.55 ± 5.56%/38.61 ± 3.66% and −2/+2; 39.08 ± 4.72%/40.07 ± 9.14%, respectively. Figure 2b, black bars). To examine the effect of a failure of glutamate transporter function, as occurs under ischemic conditions, we included a glutamate transporter blocker (TFB-TBOA) to chambers on one side of the excitotoxic insult. At a concentration that specifically blocks glial glutamate transporters (0.1 μM), or when all glutamate transporters are blocked (1 μM, not shown), cell death from spreading toxicity is exacerbated (Fig. 2c). Selective blockade of downstream NMDA receptors with MK 801 (10 μM) returns the spreading Ca^2+^ signal to baseline in the indirect chambers (Fig. 2d), and abolishes spreading toxicity (47.32 ± 7.55%, Fig. 2e). More selectively, spreading toxicity is abolished in the presence of the GluN2B antagonist, Ifenprodil (10 μM, 56.51 ± 5.05%), but not the GluN2A antagonist, NVP-AAM077 (0.5 μM, 72.09 ± 16.83%, Fig. 2f), indicating that spreading toxicity from a localized excitotoxic insult into downstream neuronal networks, occurs *via* GluN2B receptors.

### Spatiotemporal spreading neuroprotection in connected networks

**Figure 3:**
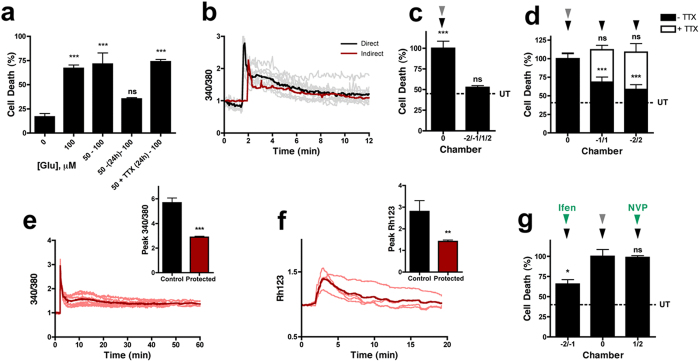
Spatiotemporal spreading neuroprotection in connected networks. **(a)** Direct preconditioning (50 GG, 1 hour) of neurons requires 24 hours (50 – (24h) – 100) to protect against an excitotoxic challenge (100 GG, 1 hour, n = 3) and is activity-dependent (blocked by 0.5 μM TTX, n = 3). **(b)** Neurons directly challenged with a preconditioning stimulus (50 GG, black trace, n = 3) undergo Ca^2+^ influx that is mirrored in downstream neurons (red trace, n = 3). Traces are representative of individual neuronal responses (light color) and the mean for all recorded cells (dark color). **(c)** Preconditioning (50 GG, 1 hour, grey arrowhead) of neurons in chamber 0 provides protection against spreading toxicity (100 GG, 1 hour, black arrowhead, n = 4) in downstream chambers (−2, −1, 1 and 2). **(d)** Neuroprotection against a direct excitotoxic insult (100 GG, 1 hour, n = 7) induced by a preconditioning stimulus (50 GG, 1 hour) in chamber 0 spreads to downstream neuronal networks (black bars). Spreading neuroprotection requires action potentials (blocked by TTX, white bars, n = 3). Protected downstream neurons exposed to 100 GG do not **(e)** undergo excitotoxicity-induced delayed Ca^2+^ deregulation and have significantly reduced peak calcium influx (n = 3) and **(f)** mitochondrial depolarization (n = 3). **(g)** Spreading neuroprotection is abolished in the presence of NVP-AAM077 (0.5 μM, n = 3) but not Ifenprodil (10 μM, n = 3). Basal cell death is indicated by the dotted line (UT). Data are expressed as mean ± S.E.M (one-way ANOVA with post hoc Tukey’s test. ‘ns’ denotes p > 0.05, *denotes p < 0.05, **denotes p < 0.01, ***denotes p < 0.001, relative to control). Experiments were performed using independent neuronal preparations.

Subtoxic insults have been reported to exert a protective preconditioning effect that protects neurons against subsequent excitotoxic challenges^13^,^14^,^15^,^16^,^17^. In keeping with this, a 1 hour treatment of 50 GG is protective against a direct excitotoxic insult (100 GG, 1 hour) delivered after 24 hours, but protection is not established immediately (Fig. 3a). This slow-onset protection requires ongoing neurotransmission, as it is blocked by TTX (Fig. 3a). Therefore, we questioned whether a preconditioning stimulus (50 GG) could also transmit protection along a neuronal network. In keeping with this, preconditioning transmits a downstream Ca^2+^ response (50 GG, Fig. 3b, black traces) in surrounding neuronal networks, although it does not reproduce the prolonged Ca^2+^ spiking activity generated by an excitotoxic insult (Fig. 1d), but does stimulate a raised Ca^2+^ level that slowly returns to baseline (Fig. 3b, red trace). Interestingly, despite preconditioning being ineffective when delivered just prior to an excitotoxic insult (Fig. 3a), it can transmit a fast acting protective signal to downstream chambers, where neurons are protected against spreading toxicity initiated from a subsequent upstream excitotoxic insult delivered immediately afterwards (Fig. 3c).

Spreading toxicity during stroke or a TBI *in vivo* is likely to be a consequence of both synaptic toxicity through hyperactivity and excitotoxicity from extracellular accumulation of glutamate^5^. To address the capacity of this fast acting network signal to also protect against a subsequent direct excitotoxic insult, a preconditioning stimulus (50 GG, 1 hour) was delivered to the central chamber, followed immediately by an excitotoxic insult (100 GG, 1 hour) to all five chambers. As seen previously (Fig. 3a,c), no protection was afforded to the directly preconditioned chamber (Fig. 3d, chamber 0). However, the spreading protective signal is capable of delivering a fast onset (<1 hour) protection against a direct excitotoxic insult (70.87 ± 16.47% and 58.29 ± 13.74% cell death in −1/+1 and −2/+2, respectively) (Fig. 3d, black bars). This fast acting network protective signaling is also activity-dependent, as it is blocked by TTX (111.53 ± 11.85% and 112.68 ± 15.51% cell death in −1/+1 and −2/+2, respectively) (Fig. 3d, white bars), and prevents excitotoxicity-induced delayed Ca^2+^ deregulation and mitochondrial depolarization (Fig. 3e,f). In contrast to the role of GluN2B NMDA receptors in spreading toxicity (Fig. 2f), GluN2A receptors are responsible for this spreading protection as it is blocked by NVP-AAM077 (0.5 μM, 98.74 ± 4.13% cell death), but not Ifenprodil (10 μM, 65.79 ± 10.78% cell death) (Fig. 3g).

**Figure 4:**
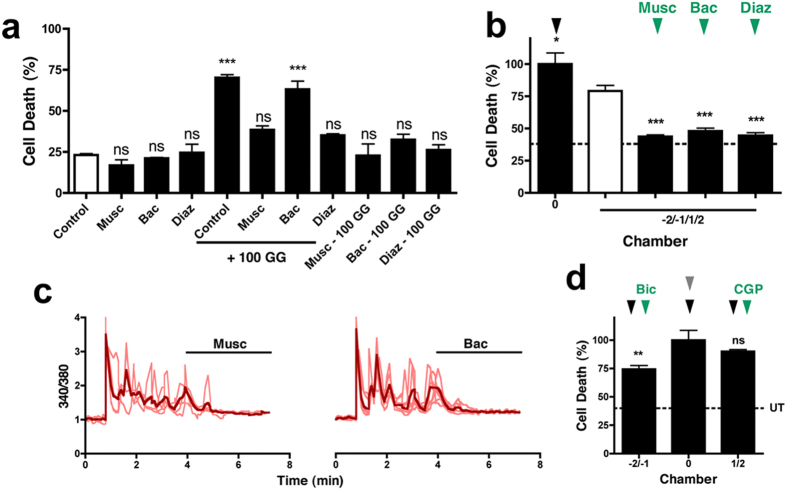
Spreading neuroprotection against a direct excitotoxic challenge requires GABA_B_ receptor function. **(a)** GABA_A_ (muscimol or diazepam) or GABA_B_ (baclofen) receptor activity attenuates glutamate excitotoxicity, although prior exposure (60 minutes) is required for GABA_B_ neuroprotection to develop (n = 3). **(b)** Spreading toxicity to downstream chambers (−2, −1, 1, 2) is blocked by enhancing GABAergic inhibition (100 μM muscimol, 5 μM diazepam, or 100 μM baclofen, n = 3). **(c)** Downstream Ca^2+^ spiking spread by an excitotoxic challenge is abolished by muscimol or baclofen (n = 3). Traces are representative of individual neuronal responses (light color) and the mean for all recorded cells (dark color). **(d)** Spreading neuroprotection from a direct excitotoxic challenge (100 GG, 1 hour) is blocked by CGP (n = 3), but not bicuculline (n = 6). Basal cell death is indicated by the dotted line (UT). Data are expressed as mean ± S.E.M (one-way ANOVA with post hoc Tukey’s test. ‘ns’ denotes p > 0.05, *denotes p < 0.05, **denotes p < 0.01, ***denotes p < 0.001, relative to control). Experiments were performed using independent neuronal preparations.

It has been reported that GABA_A_ receptor activity can attenuate NMDA-mediated excitotoxicity, whilst GABA_B_ receptor activity is ineffective^21^. In support of these findings, we observe that during an excitotoxic insult, muscimol (100 μM) and diazepam (5 μM), but not baclofen (100 μM), is neuroprotective against excitotoxicity (Fig. 4a). Interestingly however, baclofen becomes neuroprotective against excitotoxicity if present prior to (1 hour) and during the insult (Fig. 4a). We therefore investigated whether GABA_A_ and GABA_B_ receptor inhibitory activity can counter spreading toxicity. An excitotoxic insult was delivered to the central chamber (100 GG, 1 hour), while GABA_A_ or GABA_B_ receptors were stimulated (100 μM muscimol, 5 μM diazepam or 100 μM baclofen) in downstream chambers. In all cases, spreading toxicity was abolished (muscimol, 43.72 ± 0.3%, diazepam 44.53 ± 3.8%, and baclofen, 48.04 ± 3.89%) (Fig. 4b), when compared to saline-treated cells (Fig. 4b, white bar). In support of a role for increased inhibitory tone in quenching excitatory signaling and spreading toxicity, either GABA_A_ or GABA_B_ receptor activation (muscimol or baclofen, respectively) can terminate the rapid downstream Ca^2+^ spiking activity characteristic of a spreading excitotoxic challenge (Fig. 4c), further indicating that spreading hyperactivity is neurotoxic. However, protection against subsequent excitotoxicity is only blocked by GABA_B_ receptor inhibition (1 μM CGP-55845, 90.21 ± 2.24%) with little impact on protection by GABA_A_ receptor inhibition (50 μM bicuculline, 74.42 ± 12.25%) (Fig. 4d). These data suggest therefore, that a localized preconditioning stimulus initiates the recruitment of GABA_B_ receptor inhibitory neuroprotection in surrounding neurons against subsequent direct excitotoxic challenges.

### Recruiting network neuroprotection after an excitotoxic insult

**Figure 5:**
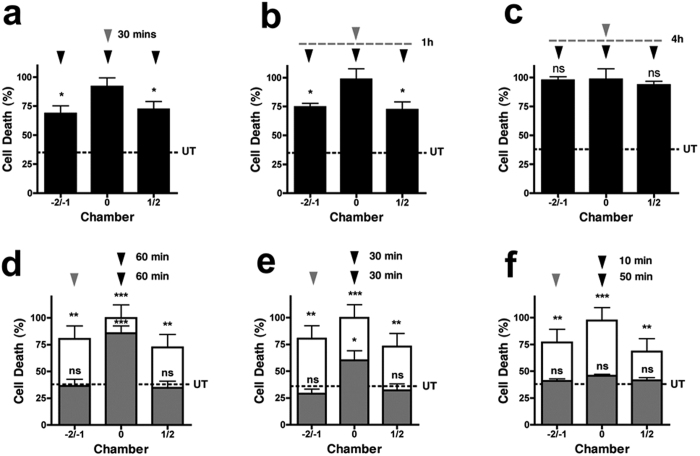
Temporal profile of spreading neuroprotection. Spreading neuroprotection occurs when the protective stimulus is reduced to 30 minutes **(a)**, or when the excitotoxic insult is delayed by 1 hour **(b)**. **(c)** Spreading neuroprotection is lost by 4 hours. **(d–f)** During an excitotoxic challenge (100 GG, black arrowhead), spreading toxicity (white bars, unconditioned response, n = 3) is blocked (grey bars, postconditioned response, n = 3) by a distal neuroprotective stimulus (50 GG, grey arrowhead) delivered after the excitotoxic challenge. In contrast, protection of neurons directly exposed to the excitotoxic challenge occurs after 10 min **(f)** and 30 min **(e)**, but not 60 min **(d)**. Basal cell death is indicated by the dotted line (UT). Data are expressed as mean ± S.E.M (one way ANOVA with post hoc Tukey’s test. ‘ns’ denotes p > 0.05, *denotes p < 0.05, **denotes p < 0.01, ***denotes p < 0.001, relative to control). Experiments were performed using independent neuronal preparations.

To investigate the temporal effectiveness of this rapid network protection, we explored whether neuroprotection could be achieved with shorter durations prior to an insult, or even post-insult. We find that an equal level of network protection is still possible when the protective stimulus (50 GG) is reduced to just 30 minutes (Fig. 5a). Furthermore, network protection is stable for at least 1 hour, as determined by delaying the excitotoxic insult (Fig. 5b). However, when the interval between the protective stimulus and toxic insult was expanded to 4 hours, no network protection is evident (Fig. 5c). This suggests that unlike preconditioning (a slow onset, persistent neuroprotection), the network protection discovered here is fast acting but transient, providing immediate protection against excitotoxic damage.

Of particular relevance to an emergency clinical situation of TBI, or following a stroke, would be the ability for a neuroprotective strategy to be effective after the onset of damage. Therefore, we investigated whether the protection could be achieved after a localised excitotoxic insult had been delivered (postconditioning). Interestingly, a protective stimulus, delivered an hour after a localized excitotoxic insult, can still afford full protection against spreading toxicity (36.44 ± 10.81% and 35.39 ± 9.67% cell death in −2/−1 and 1/2, respectively, Fig. 5d gray bars), suggesting that downstream neurons surrounding a lesion are not committed to die during this insult period. However, little protection against the direct excitotoxic challenge is evident (85.79 ± 11.48% cell death in chamber 0) in this time period. In contrast, when the protective stimulus was delivered sooner (after 30 min), significant neuroprotection is also observed (60.25 ± 15.32%) against the direct excitotoxic challenge (Fig. 5e). When the protection was delivered after just 10 minutes, full protection is observed (50.94 ± 9.17%) against the lesion in all chambers (Fig. 5f). This vanishing therapeutic window against direct ongoing excitotoxic damage most likely represents the time taken for cells to undergo terminal delayed Ca^2+^ deregulation (Fig. 1d).

**Figure 6:**
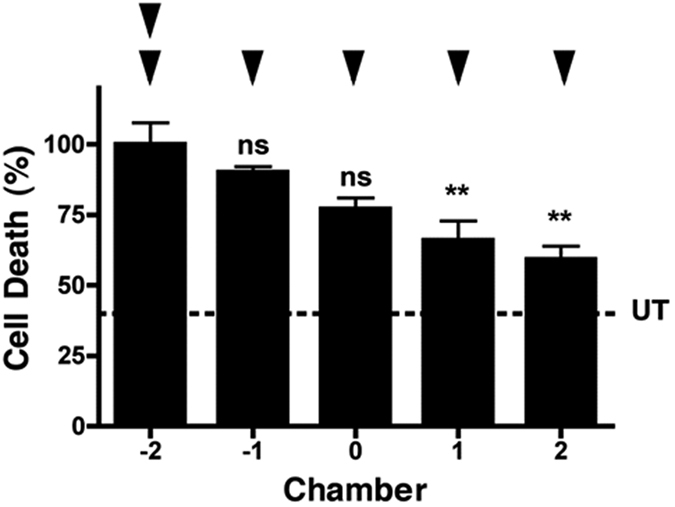
Spreading neuroprotection from an excitotoxic insult. Neuroprotection against a subsequent direct excitotoxic challenge is also spread by an excitotoxic insult (n = 3). Basal cell death is indicated by the dotted line (UT). Data are expressed as mean ± S.E.M (one way ANOVA with post hoc Tukey’s test. ‘ns’ denotes p > 0.05, **denotes p < 0.01, relative to control). Experiments were performed using independent neuronal preparations.

Interestingly, although an excitotoxic insult clearly spreads neurotoxicity, this diminishes as it progresses (Fig. 2b, white bars), suggesting either an attenuation of the toxic signal over distance, or a recruitment of neuroprotective signaling (or both). We demonstrate the existence of a spreading neuroprotective signal, emanating from an excitotoxic insult, that confers network resistance to a subsequent direct excitotoxic challenge (Fig. 6), as would occur *in vivo* with elevated glutamate levels. Therefore, neuroprotective signaling may occur naturally within neuronal networks *in vivo* in response to an insult and will ultimately dominate over spreading toxicity. This mechanism may contribute to why an excitotoxic lesion *in vivo* does not spread uncontrollably to consume the entire brain, and our study reinforces how a blanket NMDA receptor blockade will exacerbate network damage^11^ by preventing innate neuroprotective network signaling. Instead, therapeutic approaches to promote this innate spreading neuroprotective network activity may effectively terminate ongoing secondary neuronal toxicity.

## Discussion

Using hippocampal neurons cultured in multi-chambered microfluidic devices it is possible to produce environmentally-isolated neuronal populations that are synaptically interconnected^20^. Using this model, an isolated excitotoxic insult may be delivered to a neuronal network and spreading toxicity monitored. We observe that downstream non-insulted neurons may succumb to secondary spreading neuronal toxicity through GluN2B-mediated hyperactivity. In addition to the modeling of spreading toxicity, akin to a neuronal network protection racket, we demonstrate that neuronal networks offer a previously unknown, fast acting GluN2A-dependent neuroprotective signaling mechanism. This mechanism utilizes the innate capacity of surrounding neuronal networks to quench excitation, through the recruitment of GABA_B_ receptors, to provide protection against excitotoxicity. Importantly, network neuroprotection against spreading toxicity can be effectively stimulated after an excitotoxic insult has been delivered, and may identify a new therapeutic window to limit on-going brain damage in conditions of chronic (neurodegenerative disease) or acute (stroke, TBI) neuronal injury.

Stroke and TBI are leading causes of death and long term disability in adults worldwide, with patients often unable to return to work, and requiring around the clock care. Rapid therapeutic intervention is key to reducing mortality and improving the prognosis by curtailing the extent of brain damage. Beyond the lesioned area of the brain lies an area of increased vulnerability, the penumbra. Within this penumbral region, neurons may be exposed to synaptic hyperactivity^6^,^7^, toxic spreading depolarization^4^,^22^,^23^ and high levels of extracellular glutamate, leading to excitotoxicity^4^,^8^,^9^,^15^. Such a cascade of toxicity could consume entire networks, but is naturally restricted.

A number of forms of neuroprotection have been identified to date^10^,^13^,^14^,^16^,^17^,^18^,^19^ which may offer great promise in the treatment of chronic conditions, where disease progression is slow, however, protective mechanisms involving gene regulation require days to become effective (preconditioning) and so have limited therapeutic value in the emergency treatment of stroke and TBI patients. The direct approach of using NMDA receptor antagonists to block ongoing excitotoxicity has failed due to the paradoxical neuroprotective role of NMDA receptors. However, the uncoupling of NMDA receptors from cell death pathways hold great promise and may be selective against pathological NMDA receptor activity, leaving neuroprotective signalling intact^24^,^25^,^26^. To date, there are no effective strategies to prevent neurotoxicity spreading into surrounding neuronal networks. Therefore, current research has focused on promoting brain recovery^27^,^28^,^29^. Here we demonstrate a potential neuroprotective role for the use of benzodiazepines following a stroke. In contrast however, a recent meta-analysis has concluded that the potentiation of GABA_A_ receptor activity is not effective^30^. Indeed, the functional recovery of network activity is compromised by increased extrasynaptic GABA_A_ receptor activity for weeks^31^. However, inhibition of this extrasynaptic activity immediately after the onset of stroke increases the infarct size, suggesting a GABAergic neuroprotective role immediately after a stroke. This is further supported by the findings that clomethiazole is effective at reducing ischemic damage if applied rapidly^32^,^33^, but not if delayed^34^. Therefore, a strategic transient (hours) potentiation of GABA_A_ receptors^35^, followed by a prolonged (weeks) inhibition of extrasynaptic GABA_A_ receptors may be an optimal, untested, neuroprotective strategy.

This study has demonstrated that a therapeutic opportunity to curtail spreading toxicity may exist by harnessing the innate capacity of neuronal networks to block toxicity. The recruitment of this neuroprotection occurs in a timescale significantly faster (<hour) than classical preconditioning (days), where upregulation of pro-survival genes is required to boost antioxidant defence, suppress caspase activation and promote mitochondrial health^10^,^14^. Moreover, neuroprotection from preconditioning is persistent, lasting for several days^36^,^37^, while the fast-acting neuroprotection demonstrated in this study is transient, lasting for less than 4 hours. Together, this indicates that the network recruitment of neuroprotection discovered here is temporally distinct from the previously identified preconditioning pathways. However, it remains possible that normal preconditioning is tempered by the coexistence of the opposing actions of extrasynaptic NMDA receptors^38^, and a more rapid onset of preconditioning signalling may be possible.

The spreading network protection identified here, reduces deleterious Ca^2+^ influx into neurons, prevents subsequent mitochondrial depolarization and excitotoxicity-induced delayed Ca^2+^ deregulation, and is effective against both synaptic hyperactivity and extrasynaptic excitotoxicity. We demonstrate that in response to an excitotoxic lesion, both toxic and protective signals permeate through a network concomitantly, but as they progress, neuroprotection untimely dominates, and offers a mechanistic explanation of why a lesion does not spread uncontrollably to engulf the entire brain. The point at which the neuroprotective network signalling dominates may also contribute to defining the extent of the penumbral region that is susceptible to excitotoxic damage. These data are in keeping with previous studies which report a neuroprotective role for enhanced (synaptic) GluN2A and a neurotoxic role for (extrasynaptic) GluN2B receptors in mature neurons^8^,^10^,^13^,^14^.

A new potential pharmacological opportunity to limit neuronal damage is indicated by the requirement for GABA_B_ receptor function in delivering protection against subsequent excitotoxicity. However, an alternative therapeutic strategy to pharmacological manipulation may be to provide direct transcranial neuronal stimulation to connected brain regions. There is a therapeutic precedence for the use of brain stimulation in the treatment of the dysfunctional brain. A number of optical, electrical and magnetic approaches exist^28^,^39^,^40^, with electroconvulsive therapy being used for decades to treat depression and more recently proposed as a treatment for neurodegenerative disease patients^41^. Recent clinical trial evidence suggests that non-convulsive electrical stimulation may also be effective^42^, transcranial current stimulation post stroke may improve motor recovery^43^ and the extension of acupuncture to include the potential of electrostimulation (electroacupuncture) appears to have beneficial effects for acute ischemic stroke^44^.

Interestingly, in a model of Huntington disease, the synaptic: extrasynaptic NMDA receptor balance is critical in regulating mutant Huntingtin toxicity^45^ and dysregulation contributes to disease onset and progression^46^. Additionally, a recent report has identified a role for β-amyloid in increasing the expression of dendritic GluN2B, whilst decreasing the synaptic level of GluN2A in a model of Alzheimer’s disease^47^. Therefore, it may be possible that increased vulnerability to, or enhancement of, innate GluN2B-mediated spreading network toxicity and/or limitation of innate GluN2A-mediated spreading network neuroprotection, as revealed in this study, contributes to the progressive loss of neurons found in chronic neurodegenerative disease, and reflects a common mechanism of dysfunction in the brain. This would be further enhanced by the loss of synaptic contacts reported in Alzheimer’s disease^48^. It may therefore be possible, through stimulating neuronal network protective signalling, to provide a common treatment for these conditions. Future study will be required to elucidate whether the protective mechanisms identified in this study are efficacious in these chronic disease states.

In summary, we demonstrate the value of microfluidics in the study of neuronal network communication that is relevant to pathological conditions of rapid neurotoxicity (stroke, TBI) as well as conditions of network dysfunction (epileptiform activity, spreading depolarizations, neurodegenerative disease). We reveal a novel form of rapid neuroprotective signalling within networks, relying on the innate capacity of neurons to quench excitation. In previous studies, such network activity may have been overwhelmed by the initiation of global excitotoxic mechanisms. Harnessing the full potential of this innate network neuroprotective capacity may open the doors to many therapeutic opportunities.

## Materials and Methods

### Device preparation

Microfluidic devices were fabricated in polydimethylsiloxane (PDMS) using standard soft lithography techniques. Silicon masters were produced using a double layer of SU8 photoresist (3000 series, MicroChem, US) on a silicon wafer. The first layer (5 μm thick - SU8 3010) formed the microchannels (500 μm long, 10 μm wide, 40 μm pitch) connecting the five chambers (1 mm wide, 11–17 mm long), which were obtained through a second layer of resist (100 μm thick - SU8 3035). The resist was exposed through a chrome photomask (JD Photo- Tools, UK) to UV light and was developed in MicroPosit EC solvent (Rohm and Haas, US). To prevent PDMS adhesion to the resulting silicon master, the silicon surface was silanized by vapor deposition of 1H,1H,2H,2H-perfluorooctyl-trichlorosilane (Sigma Aldrich, UK) for 1 hour. PDMS was then poured onto the silicon master at a 10:1 ratio of polymer to curing agent, degassed and cured at 80 °C for at least 3 hours. The PDMS devices were then peeled from the mould, cut to the desired size, and holes were punched (4 mm diameter) to obtain the inlet and outlet wells for each chamber. PDMS devices were cleaned and irreversibly bonded to glass coverslips using oxygen plasma. Bonded devices were then washed with DI water, UV sterilized and flooded with a solution of poly-D-lysine (PDL, 15 μg/ml) for 2 hours to create a suitable adhesion layer within the chambers and microchannels. Finally, devices were washed with Neurobasal-A medium (Life Technologies) prior to cell injection.

### Neuronal cell culture

Hippocampi from 1–3 day old Sprague-Dawley rats of both sexes were dissected and minced. Tissue was suspended in filter-sterilized 1.5 mg/ml papain (Sigma Aldrich, UK) in buffer comprised of: (mM) 116 NaCl, 5.4 KCl, 26 NaHCO_3_, 1.3 NaH_2_PO4, 2 MgSO_4_, 2 CaCl_2_, 0.5 EDTA, and 25 D(+)-glucose, pH 7.4, and incubated at 37 °C for 20 minutes. Tissue was then triturated with a series of 3 flame- polished glass Pasteur pipettes of decreasing tip diameter in 10 mg/ml bovine serum albumin (Sigma Aldrich, UK) solution (in buffer as above). The resultant cell suspension was spun down and the pellet resuspended in culture medium consisting of Neurobasal-A Medium supplemented with 2% (v/v) B-27 and 2 mM L-glutamine, at a density of 3.5 × 10^6^ cells/ml. Cells were loaded into multi-chamber microfluidic devices and incubated in culture medium and maintained in a humidified atmosphere at 37 °C in 5% CO_2_. 15 μl of medium was replaced in each well every 2–3 days and experiments were performed between 15 and 20 days *in vitro* (DIV 15–20). By DIV 15, hippocampal neurons had developed a rich network of processes, expressed functional (Fura-2, AM responses) AMPA/kainate and GluN2A and GluN2B NMDA receptors, as determined by the use of AMPA/NMDA and selective antagonists (NVP-AAM077 and ifenprodil, respectively).

### Immunocytochemistry

Neuron-seeded devices were stained after DIV 15 to assess the connections between neuronal chambers, as previously described^20^. Briefly, cultures within devices were washed with phosphate buffered saline (PBS) and fixed in paraformaldehyde (4%, 10 mins). Cells were then washed in PBS and nonspecific binding prevented by incubation in a blocking solution containing fetal calf serum (5% v/v) and BSA (1% w/v) and 0.01% Triton-X100 for 30 mins. Cells were washed with PBS and primary antibodies for MAP2 (1:250 dilution) and β-III-Tubulin (neuron-specific cytoskeletal marker, 1:500 dilution) were diluted into blocking solution and incubated with cultures (1 hour). Cells were then washed with PBS, incubated with fluorescently labelled secondary antibodies, and imaged using a cooled CCD digital camera (ORCA-ER, Hamamatsu, Japan) and a 40x PL Fluotar oil-immersion objective lens on a Leica DM-IRB inverted microscope (both Leica, Germany) using Volocity Acquisition software (Improvision, UK).

### Fluorescence microscopy

Calcium imaging with Fura-2, AM (Invitrogen) was done as previously described^15^. Briefly, hippocampal neuron-seeded microfluidic devices were loaded with Fura-2, AM (3 μM) for 45 minutes at 37 °C (in the dark) in HEPES-buffered saline (HBS) comprising (mM) 135 NaCl, 5 KCl, 1 CaCl_2_, 1 MgCl_2_, 10 HEPES, and 10 D(+)-glucose (pH 7.4). Devices were washed extensively prior to experimentation, and fluorescence monitored using 340 and 380 nm excitation filters and a 535 nm emission filter. Capture rate was set at 5 seconds per time point to ensure fast changes in calcium could be detected, and data were analyzed by expressing the emission at 535 from excitation at 340 and 380 nm as a ratio, with background subtraction for each channel at each time point. Mitochondrial membrane potential was recorded using rhodamine 123 (Invitrogen). Briefly, hippocampal neuron seeded microfluidic devices were incubated with rhodamine 123 (10 μg/ml) in HBS in the dark at room temperature for 15 minutes. Devices were washed and fluorescence monitored using a 492 nm excitation filter and a 535 nm emission filter. Regions of interest containing mitochondria were selected posthoc and fluorescence intensities measured for each time point.

### Neuronal cell death

Neuron-seeded devices were treated with various pharmacological agents in HBS, as indicated. Following treatment, neurons were returned to culture media for 24 hours prior to staining with propidium iodide (PI, 20 μM) and Hoechst 33342 (Hoe, 4.5 μM) for 30 minutes. Cell death was assessed by counting both dead and live neurons in 6–8 fields of view. Cell death was expressed as a percentage of dead cells to total cell number (live + dead) and analyzed.

### Microfluidic protocol for insult delivery

To avoid cross-contamination of substances between the chambers and ensure that any toxicity spread was solely induced *via* neuronal network communication across the microchannels, both numerical simulations (COMSOL 3.5 – superimposing the laminar flow and diffusion equations) and experimental validation were performed to estimate the optimum condition of fluids to be injected in each well of the device. Briefly, prior to insult delivery, 40 μl of HBS was injected in every well (maximum volume). Subsequently, the wells of the addressed chamber were emptied and 35 μl of experimental solution (HBS + drug) was injected in the inlet well and 30 μl in the outlet well. Volumes were dispensed in 10 μl steps to minimize shear stress on the cells within the culture chambers. Every four minutes, 10 μl was withdrawn from the outlet well and 10 μl of fresh experimental solution was replaced in the inlet well. This procedure was carried out continuously for the duration of the experiment, guaranteeing no cross contamination to the adjacent chambers.

## Additional Information

**How to cite this article**: Samson, A. J. *et al*. Neuronal networks provide rapid neuroprotection against spreading toxicity. *Sci. Rep.* **6**, 33746; doi: 10.1038/srep33746 (2016).
